# Effects of simvastatin 40 mg daily on muscle and liver adverse effects in a 5-year randomized placebo-controlled trial in 20,536 high-risk people

**DOI:** 10.1186/1472-6904-9-6

**Published:** 2009-03-31

**Authors:** MRC/BHF Heart Protection Study Collaborative Group

**Affiliations:** 1 Heart Protection Study, Clinical Trial Service Unit & Epidemiological Studies Unit, Richard Doll Building, Oxford University, Old Road Campus, Roosevelt Drive, Headington, Oxford, OX3 7LF, UK

## Abstract

**Background:**

Simvastatin reduces cardiovascular mortality and morbidity but, as with other HMG-CoA reductase inhibitors, can cause significant muscle toxicity and has been associated with elevations of liver transaminases.

**Methods:**

Muscle and liver adverse effects of simvastatin 40 mg daily were evaluated in a randomized placebo-controlled trial involving 20,536 UK patients with vascular disease or diabetes (in which a substantial reduction of cardiovascular mortality and morbidity has previously been demonstrated).

**Results:**

The excess incidence of myopathy in the simvastatin group was < 0.1% over the 5 years of the trial, and there were no significant differences between the treatment groups in the incidence of serious hepatobiliary disease.

**Conclusion:**

Among the many different types of high-risk patient studied (including women, older individuals and those with low cholesterol levels), there was a very low incidence (< 0.1%) of myopathy during 5 years treatment with simvastatin 40 mg daily. The risk of hepatitis, if any, was undetectable even in this very large long-term trial. Routine monitoring of liver function tests during treatment with simvastatin 40 mg is not useful.

**Trial Registration:**

ISRCTN48489393

## Background

The HMG-CoA reductase inhibitor simvastatin is widely used to lower LDL cholesterol and reduce cardiovascular risk[1]. The substantial reductions in cardiovascular morbidity and mortality produced by lowering blood cholesterol with simvastatin were established first in hypercholesterolaemic patients with coronary heart disease (CHD)[2], and subsequently by the Heart Protection Study (HPS) and other trials, in a broad range of high risk patients with and without hypercholesterolaemia or CHD [3-7]. Large long-term randomized trials can provide valuable information on clinically relevant adverse effects of drugs that are too uncommon to be evaluated in the smaller, relatively short-term, trials upon which regulatory approval is typically based. The tolerability of simvastatin early in HPS has been reported[8], and the safety further summarised in the first report of results[3]. The lack of any detectable effect of simvastatin on the risk of non-cardiovascular mortality, haemorrhagic stroke, cancer, respiratory and neurological morbidity, and the lack of hazard in patients with diabetes or heart failure, as well as those with low blood cholesterol, have been reported in subsequent papers[4,5,9,10]. In this paper, we provide further detail about the effects on muscle and liver adverse events in HPS.

Since their introduction in the 1980s, statins have been recognised to have occasional adverse effects on muscle and liver, with the former of greater clinical importance. Few drugs have toxic effects on skeletal muscle, but all statins occasionally cause myopathy [11-13]. In this context, myopathy is generally defined as unexplained muscle pain or weakness accompanied by a creatine kinase (CK) level >10 times the upper limit of normal (ULN)[11,14]. Rhabdomyolysis is a severe form of myopathy (typically with CK >40 × ULN) that may require the patient to be hospitalised, often associated with myoglobinuria that can lead to acute renal failure and death. Though rare with all currently marketed statins, this adverse effect has been the focus of increased concern as a result of the withdrawal of cerivastatin by its manufacturer in 2001 due to a high incidence of rhabdomyolysis[15].

Treatment with lipid lowering therapy, including statins, tends to increase hepatic transaminases, but clinical hepatitis is uncommon during statin therapy[16,17]. Routine monitoring of liver function has been recommended in the prescribing information for all statins, but its usefulness has subsequently been questioned[16,18]. The size, duration and placebo control of HPS provides the opportunity to assess clinical and biochemical adverse effects on muscle and liver during treatment with simvastatin 40 mg daily, and to use this information to evaluate the value of routine monitoring of liver function tests.

## Methods

Details have been reported previously[3-6,8-10] (see also ) and are summarised below. The study was carried out in accordance with the principles of the Declaration of Helsinki and had ethics approval from Local Research Ethics Committees at each of the study sites (the study commenced prior to the advent of Multicentre Research Ethics Committees in the UK).

### Recruitment and follow-up

The HPS participants were men and women aged 40 to 80 years at high risk of cardiovascular events because of occlusive arterial disease; or diabetes mellitus; or, if male and ≥65 years, treated hypertension. People were ineligible if: their blood total cholesterol was <3.5 mmol/L (135 mg/dL); their own doctor considered statin therapy to be clearly indicated or contraindicated; they had suffered a stroke, myocardial infarction or hospitalisation for angina pectoris within the previous 6 months; they had chronic liver disease or evidence of abnormal liver function (see footnote to Table 1); severe renal disease or evidence of substantially impaired renal function (Table 1); inflammatory muscle disease or evidence of muscle problems; concurrent treatment with ciclosporin, fibrates or lipid-modifying (>1 g daily) doses of niacin; child-bearing potential; severe breathlessness (e.g. due to heart failure or COPD); life-threatening conditions other than vascular disease or diabetes (including any cancer except non-melanoma skin cancer); or any other condition that might limit long-term compliance.

**Table 1:** Reasons for patients who entered the run-in period not proceeding to the Randomisation appointment

**Reason**	**Phase of Run-in period**			
	**Placebo**	**Active**	**Unknown**	**Overall**
**Screening blood results**	**1132**	**-**	**-**	**1132**
Cholesterol <3.5 mmol/L	220	-	-	220
Liver function test abnormality*	656	-	-	656
CK>3 × ULN	78	-	-	78
Creatinine >200 μmol/L	192	-	-	192
				
**Participant vetoed or advised against by patient's physician**	**797**	**577**	**1**	**1375**
				
**Randomisation appointment cancelled**	**1100**	**1139**	**296**	**2535**
MI, angina or stroke during run-in	7	24	0	31
Died or cancer diagnosed	18	29	2	49
Myopathy	0	2	0	2
Other adverse event	193	217	0	410
Patient wishes	854	848	0	1702
Other or unknown reason	28	19	294	341
				
**Failed to attend randomisation appointment (no reason given)**	**0**	**0**	**3129**	**3129**
				
**Any of the above**	**3029**	**1716**	**3426**	**8171**

*ALT >1.5 × ULN, or >1.0 = 1.5 × ULN and AST, GGT or ALP >2 × ULN or any of AST, GGT or ALP >4 × ULN.

At the initial Screening visit, patients who appeared eligible for the trial were provided with a written description of the study and, if agreeing to participate, their informed consent sought[8]. Eligible and consenting patients had a non-fasting blood sample taken and entered a pre-randomization "run-in" treatment phase involving 4 weeks of placebo followed by 4–6 weeks of simvastatin 40 mg daily. In addition to blood lipids, CK, creatinine, alanine transaminase (ALT) and gamma-glutamyl transferase (GGT) were routinely measured by the central laboratory in the blood that had been collected at the screening visit (i.e., immediately before entering the run-in period and before any active statin treatment was started). Samples from participants with ALT >1.0 ≤ 1.5 × ULN or GGT >2 × ULN at the screening visit were also assayed for aspartate transaminase (AST) and alkaline phosphatase (ALP). Patients who entered run-in had their study treatment stopped during the placebo phase, and were not to be randomized, if their screening visit blood sample showed: (i) ALT >1.5 × ULN; (ii) ALT >1 ≤ 1.5 × ULN and GGT, AST, or ALP >2 × ULN; (iii) GGT, AST, or ALP >4 × ULN; or (vi) CK >3 × ULN, creatinine >200 μmol/L or total cholesterol <3.5 mmol/L.

As well as allowing the biochemical eligibility to be checked, the run-in period was intended to increase the likelihood that those subsequently randomized would continue taking their allocated study treatment for an extended period. In particular, participants for whom their own doctors considered statin therapy to be clearly indicated or clearly contraindicated after being provided with information about their patient's lipid levels at screening were not to be randomized. Also, patients who had (or felt they had) any adverse effects, appeared to be non-compliant or wished to drop out for any reason during run-in were not to be randomized. The use of simvastatin during run-in also allowed an indirect randomized comparison of post-randomization outcomes in participants who appeared to have a large cholesterol-lowering response to simvastatin versus those with a small response between the screening and randomization visits[3].

Participants were randomly allocated to receive simvastatin 40 mg daily or matching placebo tablets for about 5 years (and separately, using a 2 × 2 factorial design, to receive antioxidant vitamins or matching placebo capsules[19]). Randomized participants were to be seen at 4, 8 and 12 months, and then 6-monthly (with follow-up by telephone to individuals who did not attend or, alternatively, via their general practitioners). Blood samples were taken from all participants at each follow-up visit for central laboratory assay of ALT and, in any participant reporting unexplained muscle symptoms or concomitant use of a non-study statin, of CK[8]. At each follow-up visit, participants were explicitly asked by the study nurse about all episodes of unexplained muscle pain or weakness (independent of any attribution to study treatment) that had occurred since their previous visit. Information was also recorded at each follow-up visit about any suspected myocardial infarction, stroke, vascular procedure, cancer or other serious adverse event (including all hospital admissions). A serious adverse event (SAE) was defined as any new or worsening adverse condition that was fatal or life-threatening, or resulted in or prolonged inpatient hospitalization, or caused significant or persistent disability. Myopathy was considered to be a SAE irrespective of whether it resulted in hospital admission. Non-serious adverse experiences other than muscle symptoms were only recorded if they were a reason for stopping study medication.

If any SAEs were considered by the participant, study nurse or managing clinician to be possibly drug-related, or if there were potentially serious abnormalities in any safety blood tests, the participant was advised, at least temporarily, to stop the relevant study treatment. The protocol defined the following algorithms for the assessment of possible muscle or liver abnormalities during follow-up:

#### Potential muscle toxicity

Symptoms of myopathy (i.e., new unexplained muscle pain or weakness accompanied by an otherwise unexplained elevation of CK >10 × ULN[14]) would result in the study treatment (simvastatin or placebo tablets) being stopped permanently (with 3-week follow-up visits until CK reverted to normal, i.e. ≤ 3 × ULN). Creatine kinase >4 × ULN but ≤ 10 × ULN that could not be explained (e.g. by some trauma, intramuscular injection, heavy exercise, recent MI) was to be checked within about 1 week and, if repeat CK was >4 × ULN, then study treatment would be stopped temporarily. CK was to be checked again in about 6 weeks and study treatment stopped permanently if still >3 × ULN. If, on the other hand, CK was ≤ 3 × ULN, then the allocated study treatment would be started again, with 2 further early recall visits at 3-week intervals at which CK must remain ≤ 3 × ULN, otherwise the study treatment was to be stopped permanently.

#### Abnormality of liver function tests

ALT >4 × ULN was to be checked within about 1 week, while ALT >2 × ULN but ≤ 4 × ULN was to be checked within about 3 weeks. If the repeat ALT was >4 × ULN, then study treatment was to be stopped temporarily; whereas if it was >2 but ≤ 4 × ULN, ALT was to be checked again in about 3 weeks, with study treatment then stopped temporarily only if ALT remained >2 × ULN. After stopping study treatment temporarily, ALT was to be checked again in about 6 weeks and study treatment stopped permanently if still >1.5 × ULN (with 3-week follow-up visits until ALT reverted to normal, i.e., ≤ 1.5 × ULN). If, on the other hand, ALT was ≤ 1.5 × ULN, then the allocated study treatment would be started again, with 2 further early recall visits at 3-week intervals at which ALT must remain ≤ 2 × ULN, otherwise the study treatment was to be stopped permanently.

### Statistical analyses

Differences in proportions of those allocated simvastatin versus those allocated placebo were assessed using standard parametric methods and an intention-to-treat approach. No adjustment has been made for multiple hypothesis testing, but due allowance is made in the interpretation of the results.

### Role of the funding sources

The investigators were responsible for the study design, data collection, data analysis, data interpretation, and writing of the report, independently of all funding sources.

## Results

### Pre-randomization run-in period

Between July 1994 and May 1997, 63,603 people were screened in 69 UK study clinics. Of these, 32,145 patients entered the pre-randomization run-in period, and 20,536 patients were randomized. Reasons for not entering the run-in period have been reported previously[8]. Of those entering the run-in period, 8171 withdrew during the run-in period phase (Table 1) and a further 3438 attended the randomization visit but were not randomized (Table 2). Among the 8171 people who dropped out before the randomization visit, 1132 were excluded per protocol by their screening blood results and, in a further 1375 cases, the patient's physician (either their general practitioner or the local HPS collaborator) directly advised the coordinating centre or patient against proceeding to randomization. By far the most common reason (73%) for this advice was that lipid-lowering treatment was considered indicated. The randomization visit was cancelled by or on behalf of 2535 patients, and a further 3129 failed to appear for their scheduled randomization visit. Table 1 provides the reasons for dropping out before the randomization appointment subdivided by whether the report was made during the 4 week placebo or 4–6 week active phase of the run-in period. It should be noted that the placebo/active categorization is based on the date that the patient or their doctor informed the clinic or coordinating centre that they would not be continuing in the study. The actual date of any adverse event was not recorded. As the date of any such event would often be earlier (and obviously could not have been later) than the report leading to discontinuation, there is a bias in Table 1 towards more patients with adverse events being categorized in the active phase. Nevertheless, the numbers of patients dropping out are generally similar in both phases, notably for reported adverse events.

**Table 2:** Reasons for withdrawal of patients in the run-in period who attended their Randomisation appointment

**Reason**	**Overall**
Cancer diagnosed during run-in	28
MI, stroke or hospitalisation for angina during run-in	94
Lipid-lowering drug started	271
New unexplained muscle symptoms	531
Other adverse event	161
Patient wishes or long-term adherence in doubt	2992
Patient's physician discouraged participation	25
Other reasons	135
	
**Any of the above**	**3438**

Two of 32,145 patients who entered the run-in period developed myopathy while taking simvastatin and did not proceed to randomization. One of them was hospitalized with worsening renal failure, but both patients recovered. In addition, one patient had a serious rectal bleed during run-in which was attributed to an interaction between simvastatin and his anti-coagulation medication leading to a high international normalised ratio (INR). Other than these 3 patients, none of the 410 patients (Table 1) in the "other adverse event" category had a SAE attributed to study simvastatin. These "other adverse events" were predominantly relatively minor (e.g. gastrointestinal symptoms) but might have compromised long-term adherence to study medication.

Table 2 shows the reasons for not proceeding to randomization among patients attending the randomization visit. Among these 3438 participants, the most common reason (87%) for not being randomized was personal choice or problems with long-term attendance or compliance. Again, the "other adverse events" recorded were generally minor and most probably not attributable to treatment. For example, although 531 of these patients (2.2%) reported new unexplained muscle symptoms in response to an explicit enquiry (and after having been advised in the information material that simvastatin could cause muscle pain or weakness), so too did about 6% of the participants subsequently randomized to receive either simvastatin or placebo at every visit during follow-up (see below).

### Randomization and follow-up

A total of 15,454 men (75%) and 5082 women were randomized. Mean age at study entry was 64.0 years (SD 8.4), with 5806 aged at least 70 years. The mean duration of follow-up was 5.0 years for all randomized participants: 5.3 years for those who survived to the scheduled end of study treatment and about half that for those who did not (yielding 51,121 person years of follow-up for all those allocated simvastatin and 50,664 for all those allocated placebo). Baseline characteristics, compliance with study medication and changes in blood lipid levels have been presented previously [3-6].

During follow-up, only serious adverse events were routinely recorded, and many such events were study endpoints (such as major vascular events or malignancies), which have been reported previously[3-6,9,10]. Only 16 patients (9 allocated simvastatin versus 7 allocated placebo) were considered by their managing doctors, before unblinding, to have had SAEs possibly related to study simvastatin (Table 3). All of those patients had their study treatment stopped as a result. The cases of myopathy, muscle symptoms and hepatitis are discussed below, the GI haemorrhage was later attributed to colon cancer, and the 2 men who developed peripheral neuropathy (one later thought to be due to a lymphoma) were both on placebo.

**Table 3:** Serious adverse events considered (before unblinding) to be probably due to study simvastatin

**Event**	**Simvastatin-allocated**		**Placebo-allocated**	
	**(n = 10269)**		**(n = 10267)**	
Myopathy/rhabdomyolysis*	7	(0.1%)	2	(0.0%)
Muscle pain/weakness**	0	(0.0%)	1	(0.0%)
Hepatitis	1	(0.0%)	1	(0.0%)
GI haemorrhage	1	(0.0%)	0	(0.0%)
Renal failure	0	(0.0%)	1	(0.0%)
Neurological	0	(0.0%)	2	(0.0%)

* Includes 1 patient in each group taking non-study statin in addition to study treatment

** CK <10 × ULN

### Effects on muscle

Following randomization, 7 patients in the simvastatin group and 2 in the placebo group developed myopathy that was attributed to study treatment before unblinding (Table 3). Among the 7 patients in the simvastatin group, 4 were also taking CYP3A4 inhibitors (2 on erythromycin and 2 on verapamil), and one other had chronic renal failure and diabetes. Another of these patients started to take non-study simvastatin 20 mg daily about 4 years after randomization in addition to the study simvastatin 40 mg daily (i.e. a total dose of 60 mg daily) and developed myopathy 12 months later. Four of these 7 patients had CK >10,000 U/L, and were considered to have rhabdomyolysis; none developed organ damage but one patient's chronic renal failure worsened. Five of the 7 patients were hospitalized, 1 primarily for angina and 2 with other serious medical conditions (metastatic colon cancer; major urinary tract infection) that were at least partial causes of the hospitalization. All recovered from their myopathy, either after simvastatin was stopped or, in one case, after erythromycin was discontinued. Of the 2 myopathy cases in the placebo group that were attributed to study treatment, one was taking a non-study statin (cerivastatin 0.3 mg daily) in addition to their study treatment. The other placebo-allocated patient developed rhabdomyolysis after being given streptokinase for acute MI and went on to develop progressive renal failure and die after a second MI approximately 4 weeks later. An additional patient in the placebo group developed severe muscle symptoms which were considered serious and attributed to study treatment, but this was associated with a maximum measured CK of only about 5 × ULN (i.e. did not meet the pre-specified definition of myopathy).

Three additional patients allocated simvastatin and two allocated placebo developed myopathy or rhabdomyolysis that was not attributed to study treatment, perhaps because they had atypical features that made a casual relationship appear less plausible. Among the 3 patients allocated simvastatin, one developed muscle pain and weakness following a colectomy for colon cancer, but CK >10 × ULN was only detected 2 months later at the next scheduled visit when he was asymptomatic. Rhabdomyolysis was recorded on the post-mortem report (which gives coronary heart disease as the cause of death) of another patient who died following major surgery, but it is not known whether he was still taking the allocated simvastatin at that time. The third patient developed a deep vein thrombosis and thigh pain accompanied by raised CK (6800 IU/L), which was thought to be caused by fat emboli, one day after receiving tissue plasminogen activator followed by coronary angioplasty for acute MI. It is not known whether he was taking the allocated simvastatin at the time, but he later continued study treatment without further problems. Of the two placebo-allocated patients with myopathy not attributed to study treatment, one was hypothyroid and the other developed an acute compartment syndrome and rhabdomyolysis following resuscitation from a cardiac arrest.

Based on the randomized comparison, the overall incidence of myopathy irrespective of whether or not it was attributed to treatment was 0.1% (10 cases) among simvastatin-allocated patients versus 0.04% (4 cases) among placebo allocated patients, yielding an excess incidence of 0.06% over 5 years with allocation to simvastatin 40 mg daily. Muscle symptoms not caused by myopathy were very common, with no significant between-group differences: at each visit, muscle pain or weakness was reported on direct questioning by about 6% of patients allocated simvastatin and by about 6% of patients allocated placebo, with 33% of patients in each treatment group reporting muscle symptoms at least once during the study.

### Effects on liver

Following randomization, 1 patient in the simvastatin group and 1 in the placebo group developed hepatitis (both with jaundice but of unclear aetiology) that was attributed to study treatment before unblinding (Table 3). The patient allocated simvastatin was found to have ALT of 257 U/L (ULN 45 U/L) at 7 months after randomization, and simvastatin was then discontinued. The ALT remained elevated, and 16 months after randomization he was admitted to hospital with severe acute hepatitis (ALT >1000 U/L, AST = 1728 U/L [ULN 42 U/L] and bilirubin = 459 μmol/L [ULN 17 μmol/L]). Liver biopsy showed acute hepatitis with confluent necrosis, which the reviewing pathologist considered more typical of viral hepatitis than drug-induced hepatotoxicity (although viral serology and hepatic autoantibody tests were negative). The patient gradually recovered hepatic function and returned to work, although transaminases remained elevated. The case of hepatitis in the placebo group that was attributed to study treatment was subsequently found to have septicaemia.

Excluding primary or secondary hepatic malignancies (reported elsewhere[9]), there were about 200 patients in each group with liver-related SAE's. The large majority were due to biliary disease (178 [1.73%] simvastatin vs 179 [1.74%] placebo), predominantly gallstones. A total of 6 cases of hepatitis occurred in the simvastatin group and 9 in the placebo group (including the 2 cases discussed above). Ten of these patients continued their allocated study treatment after hepatitis developed, 2 stopped it for other reasons prior to the hepatitis, and 3 stopped it due to raised liver enzymes (i.e. the 2 cases considered to be due to study treatment, and a further patient with hepatitis C). It is worth noting that there were no specific exclusion criteria from HPS related to alcohol consumption.

On routine monitoring at each follow-up visit, ALT >3 × ULN (i.e. the "traditional" cut-point for a significant transaminase elevation[20]) was found in 77 patients allocated simvastatin and 65 allocated placebo (0.75% vs 0.63%; P = 0.35) on at least one occasion during an average of 5 years of follow-up. There appeared to be a significant difference between the treatment groups early after randomization: at the first (4 month) follow-up visit, ALT >3 × ULN was found in 17 simvastatin vs 4 placebo patients (0.17% vs 0.04%; P < 0.01). Such ALT elevations were re-checked according to the pre-specified algorithm described above. Most of these elevations were not confirmed at these early recall visits despite continued treatment: overall, ALT >3 × ULN on repeat testing was found in only 21 patients in the simvastatin group compared to 9 in the placebo group (0.20% vs 0.09%; P = 0.045); and of these, only 1 vs 0 were diagnosed clinically as hepatitis. Of the elevations detected at the first follow-up visit, 8 vs 0 were confirmed on repeat testing, but none developed liver disease. Six of these 8 were permanently discontinued from treatment according to the study algorithm, so it is not known whether they would have developed clinically apparent liver toxicity had they continued taking simvastatin.

### Reasons for stopping allocated simvastatin or placebo

Compared to the simvastatin group, more patients in the placebo group stopped taking study medication (33.5% vs 43.1%; p < 0.0001: Table 4). This difference was chiefly attributable to more patients allocated placebo starting a non-study statin (3.5% vs 13.6%; p < 0.0001), with very similar numbers of patients stopping for other reasons (28.3% simvastatin vs 27.8% placebo; p = 0.45). Discontinuation was recorded by the clinic nurses as being due to liver or muscle enzyme abnormalities in 57 patients in the simvastatin group and 46 patients in the placebo group (P = 0.32). Such discontinuations could result either from application of the protocol-defined algorithms for liver function test or CK abnormalities measured within the study, or from enzyme abnormalities detected outside the study. Because this reason for stopping was recorded using a single check box on the clinic form, it is not possible unequivocally to separate the liver versus muscle enzyme abnormality discontinuations. However, it has been possible to subdivide these patients using retrospectively defined cut-points of CK (>4 × ULN), ALT (>1.5 × ULN) and GGT (>2 × ULN) measured during their previous 4 study visits. Using these cut-points, muscle enzymes were elevated (with or without elevated liver enzymes) in 11 simvastatin versus 2 placebo patients (0.11% vs 0.02%; P = 0.03), liver enzymes alone were elevated in 42 versus 36 (0.41% vs 0.35%; P = 0.57), and neither were elevated in 4 vs 8. Five simvastatin vs 0 placebo patients had study treatment stopped with CK >4 × ULN recorded at one or more of these previous visits but without achieving the criteria for a diagnosis of myopathy (i.e. CK >10 × ULN with unexplained muscle pain or weakness).

**Table 4:** Reasons for randomised patients stopping study simvastatin/placebo tablets

**Reason(s) given**	**Simvastatin-allocated** **(n = 10269)**		**Placebo-allocated** **(n = 10267)**		
Patient wishes	2186	(21.3%)	2113	(20.6%)	
Unable or unwilling to attend clinic	757	(7.4%)	745	(7.3%)	
Non-study statin started	364	(3.5%)	1401	(13.6%)	
Other contraindicated drug started	11	(0.1%)	50	(0.5%)	
Poor compliance	88	(0.9%)	120	(1.2%)	
Raised liver or muscle enzymes**	57	(0.6%)	46	(0.4%)	
Raised liver enzymes	46	(0.4%)	36	(0.4%)	
Raised muscle enzymes	11	(0.1%)	2	(0.0%)	
Muscle pain or weakness	60	(0.6%)	62	(0.6%)	
Medical advice	158	(1.5%)	206	(2.0%)	
Medical diagnosis/treatment/investigation	143	(1.4%)	160	(1.6%)	
Non-specific adverse events	110	(1.1%)	126	(1.2%)	
Other adverse events	229	(2.2%)	225	(2.2%)	
Gastrointestinal	114	(1.1%)	104	(1.0%)	
Psychological/psychiatric	49	(0.5%)	54	(0.5%)	
Rash or skin	22	(0.2%)	22	(0.2%)	
Neurological	10	(0.1%)	14	(0.1%)	
Other	44	(0.4%)	43	(0.4%)	
Other or family reasons	149	(1.5%)	141	(1.4%)	
					
Any of the above*	**3440**	**(33.5%)**	**4424**	**(43.1%)**	**P = 0.0000**

* Patient may stop study treatment for more than one reason

** Includes 6 vs 2 myopathy cases (the remaining myopathy patients had no further visits) and 2 vs 1 hepatitis cases

## Discussion

The randomization and systematic follow-up of over 20,000 patients in HPS has allowed the reliable placebo-controlled assessment of the clinical and biochemical effects on muscle and liver of about 5 years of simvastatin 40 mg daily. It provides further evidence of the safety of this treatment regimen, with an incidence of myopathy that was less than 0.1% over 5 years. Simvastatin 40 mg daily did produce an excess of ALT >3 × ULN of about 0.1% in the first few months after randomization, but there was no evidence that this resulted in subsequent liver disease. Prior to randomization, many potential drop-outs were excluded during the run-in period or at the randomization visit in order to improve the statistical sensitivity of HPS to assess the efficacy of prolonged simvastatin [21]. The pre-randomization run-in period involved 4 weeks of treatment with placebo simvastatin followed by 4–6 weeks of simvastatin 40 mg daily (with the active antioxidant mixture given throughout the run-in period), which raises questions about the generalisability of the safety analyses because patients may have withdrawn during the run-in due to adverse effects caused by simvastatin. The potential impact of the run-in period on the assessment of safety in HPS is considered below.

### Effects on muscle

Approximately one third of the randomized participants reported muscle symptoms at some time during follow-up, but there were no significant differences between the treatment groups at any time or when subdivided by age, gender or pre-existing disease. This high rate of reported muscle symptoms may reflect a high background rate of musculoskeletal problems in middle and old age (mean age of the study population was 64 at randomization), but it may also reflect the advice given during the informed consent process that simvastatin can cause muscle pain and weakness which was reinforced by regular direct questioning at each follow-up visit. Using the pre-specified definition for myopathy (i.e. unexplained muscle pain or weakness accompanied by CK >10 × ULN[11,14]) there were 10 (0.10%) cases among patients randomly allocated simvastatin 40 mg daily and 4 (0.04%) cases in the placebo group (not all of which was attributed to study treatment). This difference is not statistically significant, but – given that myopathy is a well-established adverse effect of simvastatin (and of other statins, particularly at higher doses [15,22]) and the cases in the placebo group had predisposing factors – it is likely that some of this excess was caused by simvastatin.

Among 32,145 patients who started the run-in period for HPS, only 0.2% (78) of patients with CK >3 × ULN in blood collected at the screening visit were withdrawn during the placebo phase, and the impact of their exclusions seems likely to be small. During the active run-in phase on simvastatin, 2 (< 0.01%) cases of myopathy were recorded. One of these patients was attending a renal clinic for pre-existing renal impairment which was an exclusion criterion since it can pre-dispose to myopathy[22]. Patients were asked about muscle symptoms at the randomization visit and were not to be randomized if they reported new unexplained muscle pain. But, given the lack of difference between the simvastatin and placebo groups in post-randomization reports of muscle symptoms, it seems unlikely that exclusion of the 531 patients who reported new unexplained muscle pain (2.2% of those attending) at their randomization visit would have materially reduced the subsequent incidence of statin-induced myopathy. Moreover, HPS still provides a completely unbiased assessment of the risk of myopathy with simvastatin 40 mg daily during 5 years of treatment among patients who present with CK <3 × ULN and can tolerate treatment for about one month. In that regard, HPS reflects real-life since the incidence of myopathy in patients who do not (or believe they do not) tolerate the drug, and so will not be exposed to long-term therapy, is moot.

### Effects on liver

Prescribing information for simvastatin (and other statins) has typically recommended routine liver function monitoring, even though – despite over 20 years of clinical investigation and widespread use – there is still no good evidence that the drug is hepatotoxic. There is no question that statin therapy can increase blood concentrations of hepatic transaminases, but it has never been shown that such increases progress to clinical hepatitis if treatment continues. Moreover, these statin-induced transaminase elevations are not accompanied by increases in bilirubin, which the US Food and Drug Administration (FDA) considers to be the hallmark of a drug likely to produce significant liver toxicity[20]. Furthermore, all drugs that reduce LDL cholesterol levels appreciably (including bile acid sequestrants, which are not absorbed, and ezetimibe) produce small increases in the blood levels of transaminases[16], suggesting that the increases seen with simvastatin and other statins are (at least in part) a secondary effect of lowering LDL cholesterol.

Following randomization in HPS, hepatitis was recorded in a total of 6 patients allocated simvastatin and 9 allocated placebo during an average of 5 years of follow-up. Hence there was no significant evidence that simvastatin 40 mg daily materially increased the risk of clinical hepatitis. Liver function monitoring during HPS was intensive, with ALT (which is considered to be most sensitive to statin-induced changes[20]) measured at every visit, so it cannot be proved that such monitoring did not prevent some patients from proceeding to hepatitis. But, this seems unlikely to have had much impact, since there was only a small difference between the treatment groups in ALT >3 × ULN at the first 4 month follow-up visit (17 [0.17%] simvastatin vs 4 [0.04%] placebo) and no apparent difference thereafter. Similarly, among the 4444 patients in the Scandinavian Simvastatin Survival Study (4S), there was only a small difference between the treatment groups in ALT >3 × ULN which was confined to the first year of treatment (20 [0.90%] allocated simvastatin 20–40 mg daily vs 8 [0.36%] allocated placebo)[23]. By contrast, drugs that are hepatotoxic typically produce marked transaminase elevations at a rate that is much higher than the incidence of clinical hepatitis[24].

Irrespective of whether the ALT elevations in HPS occurred early or late following randomization, most were not found to have persisted on repeat testing despite continuation of the simvastatin treatment, suggesting that they are not likely to reflect a clinically relevant adverse drug effect. Whether the residuum of persistent elevations has any clinical meaning is uncertain, but the absolute excess of patients discontinued due to liver enzyme abnormalities in HPS was very small (about 1 per 1000) and not statistically significant. A comparably small, and non-significant, excess of study treatment discontinuations due to liver enzyme abnormalities was observed in 4S (8 [0.36%] simvastatin vs 5 [0.22%] placebo)[23].

Among 32,145 patients who started the run-in period for HPS, 2% (656) of patients with abnormal liver function tests in blood collected at the screening visit were withdrawn during the placebo phase. No patient who attended the randomization visit failed to proceed to randomization because of an abnormal liver function test after taking 4–6 weeks of active simvastatin during the run-in period, but 6 randomized patients were subsequently found to have ALT >3 × ULN in blood collected at the randomization visit. None of these elevations persisted on repeat testing when these patients were no longer taking simvastatin (since all 6 had, by chance, been allocated placebo) and none was discontinued from study treatment. Consequently, the pre-randomization run-in period in HPS is not likely to have biased the assessment of the effects of 5 years of treatment with simvastatin 40 mg daily on the risks of clinical hepatitis. Consequently, most regulatory agencies have now allowed the recommendations in the prescribing information for simvastatin to indicate that routine liver monitoring is not needed for doses of simvastatin up to and including 40 mg daily. It remains uncertain whether, these conclusions can be extrapolated to simvastatin 80 mg daily (the maximal recommended dose of this drug), and this is now being assessed as part of a large-scale direct randomized comparison of 80 mg versus 20 mg simvastatin daily[22,25].

## Conclusion

The present results provide further evidence of the safety of long-term treatment with simvastatin 40 mg daily. The risk of myopathy was rare, with an incidence of less than 0.1% over 5 years, and there was no evidence that simvastatin 40 mg daily causes clinical hepatitis. Routine monitoring of liver function tests during treatment with simvastatin at daily doses up to 40 mg did not appear to be of value, at least in patients with liver function tests that were normal prior to starting treatment.

## Competing interests

The Clinical Trial Service Unit has a staff policy of not accepting honoraria or other payments from the pharmaceutical industry, except for the reimbursement of costs to participate in scientific meetings. Coordinating centre members of the writing committee (J Armitage, R Collins, L Bowman, S Parish) have, therefore, only had such costs reimbursed. J Tobert is a retired employee of Merck and Co, holds stock options granted when he was an employee, and has provided consultant services to Merck and other companies with lipid-lowering products.

## Authors' contributions

MRC/BHF Heart Protection Study Collaborative Group

*Writing Committee*-Jane Armitage, Louise Bowman, Rory Collins, Sarah Parish and Jonathan Tobert

JA was involved in data collection, analysis and interpretation, and drafted the manuscript. LB was involved in data collection, analysis and interpretation, and drafting of the manuscript. RC conceived and designed the study, was involved in data collection, analysis and interpretation, and drafting of the manuscript. SP was involved in data collection, analysis, validation and interpretation. JT was involved in the study design and interpretation, and drafted the manuscript. All authors provided comments on and approved the final manuscript.

*Steering Committee*-R Collins (principal investigator), T Meade (chairman), P Sleight (vice-chairman), J Armitage (clinical coordinator), S Parish and R Peto (statisticians), L Youngman (laboratory director), M Buxton, D de Bono (deceased), C George, J Fuller, A Keech, A Mansfield, B Pentecost, D Simpson, C Warlow; J McNamara and L O'Toole (MRC observers).

*Data Monitoring Committee*-R Doll (chairman, deceased), L Wilhelmsen (vice-chairman), K M Fox, C Hill, P Sandercock.

*Collaborators*-listed in reference 3.

## Pre-publication history

The pre-publication history for this paper can be accessed here:


